# Serum Vitamin D, Folate and Fatty Acid Levels in Children with Autism Spectrum Disorders: A Systematic Review and Meta-Analysis

**DOI:** 10.1007/s10803-021-05335-8

**Published:** 2021-11-03

**Authors:** Maria Carmen Gallardo-Carrasco, José Antonio Jiménez-Barbero, María del Mar Bravo-Pastor, David Martin-Castillo, María Sánchez-Muñoz

**Affiliations:** 1 “Los Arcos” Mental Health Center, Paraje Torre Octavio, 54, PC: 30739 Pozo Aledo, Murcia, Spain; 2 Department of Nursing, University of Murcia, Espinardo Campus, Building 23, PC: 30100 Murcia, Spain; 3 Nursing Department, University School of Nursing of Cartagena, University of Murcia, , Campus de Cartagena, Paseo Alfonso XIII, 61, PC: 30203 Cartagena, Murcia, Spain; 4 Department of Nursing, University of Murcia, Espinardo Campus, Building 23, PC: 30100 Murcia, Spain

**Keywords:** Autism, autism spectrum disorders, Fatty acids, Vitamin D, Folic acid levels

## Abstract

Vitamin and fatty acid deficiency in children diagnosed with autism has been linked to the etiology and course of the disease but the results have been inconsistent. In our work, we present a narrative review, which includes 20 observational studies that provide data on the blood levels of vitamin D, folate, or fatty acids of children diagnosed with ASD (Autism Spectrum Disorder—AG group), and of a control group (children without this disorder—CG group). The main characteristics and results are presented in a summary table. Of the 20 above-mentioned studies, a meta-analysis of vitamin D and folate levels was carried out in 14 of them, with a total of 2269 children (AG = 1159, CG = 1110). Vitamin D levels were lower in AG compared to CG: SMD, 95% CI = − 0.83 [− 1.15, − 0.50]. In terms of folate levels, a total of 299 children (AG = 148, CG = 151) were analyzed, finding no significant differences with the control group: SMD, 95% CI = − 0.16 [− 0.63, 0.32]. Only one study that provided data on fatty acids in children with ASD was included in the review although it was not possible to include it in the meta-analysis. We conclude that the nutritional status (vitamin and fatty acid levels) of patients diagnosed with ASD should be taken into account, as correct adjustment of these levels—may produce an improvement in the course of the disease and could also reduce the risk of its development.

**Supplementary Information:**

The online version contains supplementary material available at 10.1007/s10803-021-05335-8.

## Introduction

Autism is a neurodevelopmental disorder initially described by Kanner (1943), characterized by alterations in social and verbal communication, limited and repetitive interests, and currently considered a serious mental disorder (Lord et al., 2018).

The global prevalence trend is a marked rise (Kerub et al., 2021; Málaga et al., 2019; Tioleco et al., 2021), although it is worth noting the great variability between the data depending on the country of the study (Belinchón et al., 2010; Fuentes et al., 2020; Málaga et al., 2019; Marín et al., 2016; Matson & Kozlowski, 2011; Nevison & Parker, 2020). In the US, according to the latest update from the Center for Disease Control and Prevention (CDC), the prevalence of ASD (Autism Spectrum Disorder) was 18.5 per 1000 (1:54) 8-year-olds, and ASD was 4.3 times more prevalent among boys than among girls. This prevalence varied by birthplace, from 13.1 (Colorado) to 31.4 (New Jersey) (Maenner et al., 2020). However, in Spain, the prevalence ranges from 15.5/1000 (3–4 years) to 10/1000 (10–11 years), with a ratio of 1:64 (3–4 years) to 1:100 (10–11 years) (Morales-Hidalgo et al., 2018).

The etiology of ASD remains little known, although different areas might be related (Kheirouri & Alizadeh, 2020). Thus, for example, the influence of genetics, inflammatory processes, autoimmune disorders, oxidative stress, neurotransmitters, and environmental factors has been demonstrated (Chen et al., 2021; Kinney et al., 2010; Thorsen et al., 2021). Similarly, prenatal and perinatal events, such as maternal infection, medication, and parents’ age have been identified as possible risk factors for autism and have received considerable attention (Guo et al., 2020).

Different studies have reported the finding of differences in blood levels of vitamins (D_2_,_3_ and B_9_) and fatty acids in children diagnosed with autism, compared to children without this disorder (Du et al., 2015). We note that vitamin D, ergocalciferol (D2) or colecalciferol (D3), is a fat-soluble vitamin present in the diet in limited amounts, which is mainly obtained from skin exposure to UVB radiation (De Luca, 2004). This differs from folate or B9, a water-soluble vitamin obtained through the diet. According to the indications of the “Endocrine Society”, vitamin D deficiency is defined as a serum concentration of 25 (OH) D < 20 ng/mL, insufficiency as 21–29 ng/ml, and sufficiency as at least 30 ng/ml for adequate health (Holick, 2017).

The relationship between vitamin D deficiency and ASD is not entirely clear, and there are numerous studies, some of them quite recent, that relate deficient levels of these biochemical parameters in children diagnosed with ASD, compared to children without neurodegenerative pathologies (Agostoni et al., 2017; Arastoo et al., 2018; Bala et al., 2016; Bozzatello et al., 2016; Eyles et al., 2013; Farid et al., 2016; Frye et al., 2018; Garipardic et al., 2017; Guo et al., 2019; Liu et al., 2016; Parletta et al., 2016).

In this sense, rigid behavior, as children with ASD usually pose difficulty accepting changes in food textures or incorporating new foods into their daily diet (Cermak et al., 2010; Esteban-Figuerola et al., 2019; Sánchez et al., 2015; Yule et al., 2020), and difficulty relating to others, as well as sharing outdoor spaces with others, thus hindering sun exposure (Liu et al., 2015; Wang et al., 2021), have been described as causes that justify the pattern of vitamin D deficiency in children with ASD. Other authors propose dysfunction in the gut microbiota and gut-microbiota-brain relationship (Ogbu et al., 2020), as well as a decrease in vitamin D receptors (Altun et al., 2018).

### The Present Study

The bibliography review has shown evidence of the relationship between vitamin D and folate levels and the diagnosis of autism in children (Altun et al., 2018; Frye et al., 2018; Garipardic et al., 2017; Gong et al., 2014; Mazahery et al., 2016; Sun et al., 2016). In fact, a recent meta-analysis assessed the relationship between vitamin D levels and autism, including studies published through November 2019 (Wang et al., 2021). Our work, however, represents an update and extension of previous studies, as it includes studies published until December 2020 that analyze vitamin D levels, folate, and fatty acids in children diagnosed with ASD versus children without this diagnosis.

The main objective is therefore to study the differences in blood levels of vitamins and fatty acids between children diagnosed with ASD compared to children without this diagnosis. For this purpose, a meta-analysis of observational studies was carried out that established comparisons between the values obtained in children diagnosed with ASD compared to children without this disorder. In addition, a narrative review is offered which considered all the studies that met the inclusion criteria but did not provide sufficient data to perform the meta-analysis.

Following the PRISMA 2020 (Page et al., 2021) criteria for research question formulation, we drew on the following approach: Are there significant differences in the reported levels of vitamin D, folate, and fatty acids in children diagnosed with ASD compared to children without this diagnosis?

## Method

The protocol used in this systematic review follows the recommendations of (Page et al., 2021). Similarly, the methods used in the review were specified in advance and documented in a protocol, which is available online: (https://www.crd.york.ac.uk/PROSPEROFILES/125932_PROTOCOL_225905.pdf).

### Inclusion and Exclusion Criteria

According to the research question posed, the studies were included in the review if they met the following criteria: (a) studies should compare blood levels of vitamin D and/or fatty acids and/or folate in children diagnosed with ASD with the values in children without this disorder; (b) the participants’ age was between 0 and 18 years; (c) studies should have been published between 2014 and 2020; (d) ASD was diagnosed using the Diagnostic and Statistical Manual of Mental Disorders, fifth edition DSM-V (APA, 2014); (e) the age of the control group must range from 0 to 18 years and they should not have any neurodevelopmental pathology.

The outcome measures included in the study were:

1. Vitamin D levels, 25-hydroxyvitamin D, or 25-OH vitamin D, obtained through blood sample and analyzed in the laboratory by chemiluminescence immunoassay and expressed as ng/ml.
2. Levels of folic acid, folate, folacin, pteroylmonoglutamic acid, or vitamin B_9_, obtained through blood sample and analyzed in the laboratory with an enzyme immunoassay technique (ELISA).
3. Fatty acid levels. The three main omega-3 fatty acids measured are: alpha-linolenic acid (ALA), eicosapentaenoic acid (EPA), and docosahexaenoic acid (DHA). Obtained through blood sample and analyzed in the laboratory by capillary gas chromatography, with flame ionization detection.

Exclusion criteria were: (a) articles that did not propose among their objectives the measurement of blood levels of vitamin D, folate, and fatty acids in people diagnosed with ASD; (b) that the study population was over 18 years of age; (c) secondary studies (narrative or systematic reviews).

### Search Strategy

A systematic search in the following electronic databases was conducted: Medline, Cochrane, Pubmed, PsycINFO, and Web of Science (WOS). The main descriptors used were: “*25-OH*” AND “*cholecalciferol*” OR *“vitamin*” AND “*D3*” OR “*vitamin D3*” AND “*autistic disorder*” OR “*autism*” OR “*ASD*”. The last search was performed on January 10, 2021, in the WOS. The complete strategy used can be found in “Addendum 1”.

The search was conducted by two independent researchers who made lists of potentially eligible articles. These lists were subsequently agreed upon, and any disagreements were resolved through the intervention of a third reviewer. To reduce unplanned duplication of comments and to provide transparency to the review process, as well as to minimize reporting bias (Booth et al., 2013), this study was recorded in PROSPERO (International Prospective Register of Ongoing Systematic Reviews) http://www.crd.york.ac.uk/prospero since its initiation; Registry No: CRD4225905.

### Selection of the Studies

Study selection was carried out in two phases, following the indications of the PRISMA statement (Moher et al., 2015):

- In the first phase, two reviewers independently examined potentially eligible studies by reading titles and abstracts, following a previously made checklist, which included the selection criteria described in the protocol. Listings of preselected articles were subsequently agreed upon, resolving discrepancies by discussion.

- In the second phase, two reviewers independently read the full text of the studies preselected in the previous phase, again creating two lists of potentially eligible articles. Disagreements were resolved through discussion, and a third reviewer was required to intervene when no consensus was reached. The complete texts of the accepted articles were carefully read, and their lists of bibliographic references were examined to identify possible relevant articles that had not been located in the initial search.

### Analysis of Bias Risk

The selected studies were subsequently submitted to risk-of-bias analysis, which was performed by two independent reviewers. The instrument used by these reviewers was the STROBE initiative statement for observational studies consisting of 22 items (Von Elm et al., 2007). 1 point per item was awarded, using the following criteria: 1 point if the item was fulfilled, 0.5 points if the item was partially fulfilled, and 0 points if the item was not fulfilled at all. The cut-off point for the eligibility of the studies was established at the mean value of each scale, that is, the article had to exceed 50% of items on the evaluation scale to be included in the systematic review. In cases where no consensus was found for the acceptability of an article, a third reviewer was consulted. Finally, interjudge reliability was calculated using intraclass correlation analysis.

### Tabulation and Data Analysis

The studies included in our study were coded in an Excel database by the first author. The coding was reviewed by the second and third authors, and doubts were resolved through discussion among all the authors. Subsequently, summary tables were created in which the data of each selected study were recorded according to the following categories: date and country of study, research objective, size and age of sample used, study design, outcome measures, and significant results.

### Data Synthesis

First, a narrative synthesis of the included studies was carried out, based on the outcome measures: folate levels, vitamin D, and fatty acid levels. An ad-hoc table was developed with the main characteristics and results (Table 1).

**Table 1:** Studies included in the narrative review

Author/country	Objective	Sample Size/%sex ASD	Age (Years)	Duration	Outcome measures	Significant outcomes
(Uğur & Gürkan, 2014) Turkey	-Investigate serum levels of vitamin D, calcium (Ca), phosphorus (P), alkaline phosphatase (ALP) and folate in 54 young children, ages 3 to 8, with autism spectrum disorders (ASD) and in 54 normal control children paired in age and sex	54 ASD 87% male 54 CG	3–8	7 months	-Vitamin D levels -Folate levels	-They did not establish significant differences between vitamin D and folate levels between ASD and CG group: vitamin D: ASD: 25.12 ± 11.28; CG: 21.11 ± 9.65 Folate: ASD: 12.46 ± 5.84; CG: 12.68 ± 4.37
(Gong et al., 2014) China	-Evaluate vitamin D levels in sample of Chinese children with ASD	48 ASD 40% male 48 CG	3–8	12 months	-Vitamin D levels	-Vitamin D levels obtained were lower in the ASD group than in healthy controls: 19.9 ± 3.8 vs 22.6 ± 4.5
(Bener et al., 2014) Qatar	-Determine the association between vitamin D and autism -Determine difference in vitamin D levels between controls and autistic children	254 ASD 65% male 254 CG	3–8	2 years	-Vitamin D levels	-Lower vitamin D levels in ASD group than in CG. 18.39 ± 8.2 vs 21.59 ± 8.4
(Fernell et al., 2015) Sweden	-Address the emerging hypothesis that low levels of vitamin D increase the risk of ASD	29 ASD 89% male 29 CG	14–32 months	6 months	-Vitamin D levels	-Vitamin D deficiency more accentuated in ASD group than in CG. 24(19.6) vs 31.9(27.7)
(Farid et al., 2016) Egypt	-Determine dietary intake of vitamin D and sun exposure and its impact on vitamin D level	49 ASD 81% male 40 CG	3–15 years	6 months	-Vitamin D levels	-Lower vitamin D levels in ASD group than in CG. 46.5(14–120) vs 70.89 (16–149)
(Parletta et al., 2016) Australia	-Compare PUFA levels in erythrocytes in children with ADHD, ASD, and typical developing controls, and investigate correlations between PUFA levels and respective symptoms	85ASD 80% male 79 CG 401TDAH	3–17 years	March 2004 December 2010	-Blood levels of fatty acids: DHA, AA, EPA + DHA	-Lower levels of EPA, DHA, and AA in ASD and ADHD versus CG. DHA: ASD: 0.557 ± 0.524; CG: 1.798 ± 0.5894 AA: ASD: 0.557 ± 0.524: 0.851 ± 0.564; CG: 4.715 ± 1.020. EPA + DHA: ASD; CG: 10,522 ± 2056
(Bala et al., 2016) Turkey	-Analyze thyroid hormones and antibodies, ferritin, vitamins B12 and D, adrenal and gonadal steroid levels, and celiac antibodies in children diagnosed with ADHD and ASD	16 ASD 62.5% male 27 CG 34 TDAH	2–17 years	February 2014 July 2014	-Vitamin D levels -Folate levels	-Vitamin D levels lower in ASD than in CG. ASD; CG: 28.73 ± 9.04 -Slightly lower folate levels in CG; CG: 8.52 ± 3.75; ASD
(Liu et al., 2016) China	-Determine whether growth, eating behaviors, and gastrointestinal symptoms of children with ASD differ from those of controls; whether biochemical nutrition index levels are lower in children with ASD compared to Chinese standards; and possible relationship between nutritional status (vitamin A (VA), VD, vitamin B12 (VB12), ferritin (FER), hemoglobin (Hb), and folate (FOL) and ASD symptoms	154 ASD 91.6% male 73 CG	3–7 years	August 2013 October 2014	-Vitamin D levels -Folate levels	-Vitamin D values in ASD group: 22.55 ± 7.43 -Folate levels in ASD: 9.07 ± 3.84 -They did not establish significant results between levels and ASD symptoms
(Bener et al., 2017) Germany	-Investigate iron deficiency anemia and vitamin D deficiency in children with autism and assess the importance of risk factors (determinants)	308 ASD 43.7% male 308 CG	8 years	June2011 May2014	-Vitamin D levels	-Vitamin D values in ASD lower than in CG. ASD: 18.79 ± 8.35; CG: 22.18 ± 9.00
(Garipardic et al., 2017) Turkey	-Evaluate mean platelet volume (MPV) values in children with ADHD and ASD to determine the risk of cardiovascular disease in these 2 groups of disorders	18 ASD 61.1% male 25 CG 36 ADHD	2–18 years	February 2014 July 2014	-Vitamin D levels -Folate levels	-Vitamin D levels in ASD were lower than in CG. ASD: 14.3 ± 7.25, CG: 29.42 ± 9.07 -Folate values in ASD: 9.09 ± 3.91; CG: 8.43 ± 3.85. No significant relationships were established in this hematological parameter
(Altun et al., 2018) Turkey	-Examine serum levels of vitamin D, vitamin D receptor (VDR), homocysteine, vitamin B6, vitamin B12, and folate in ASD	60ASD 86.6% male 45CG	3–12 years	March September Year N/A	-Vitamin D levels -Folate levels	-Lower vitamin D values in ASD than in CG. ASD: 13.79 ± 1.03; CG: 16.58 ± 1.06 -Folate values lower in ASD than in CG. ASD: 121.16 ± 8.04; CG: 172.31 ± 17.19
(Wu et al., 2018) China	-Estimate the prevalence of ASD in Chinese children 3 years of age and examine the association between neonatal vitamin D status and the risk of ASD	310 ASD 77.4% male 1240 CG	3 years	2008–2010	-Vitamin D levels	-Vitamin D levels in ASD group significantly lower than in CG. ASD: 17.6 Z: 11.997; CG: 40.2 Z: 12.986
(El-Ansary et al., 2018) Saudi Arabia	-Determine possible relationship between vitamin D levels, proven biomarkers, and the presence and severity of ASD	28 ASD 100% male 27 CG	7 years	N/A	-Vitamin D levels	-Vitamin D levels in ASD were lower than in CG. ASD: 95.63 ± 26.63; CG: 140.43 ± 17.68
(Arastoo et al., 2018) Iran	-Assess the serum level of vitamin D among children with ASD in the city of Ahvaz, Iran	62 ASD 84% male 31 CG	9 years	1 year	-Vitamin D levels	-Establish significant differences between ASD and CG levels. ASD: 9.04 ± 4.14, CG: 15.25 ± 7.89
(Bičíková et al., 2019) Czech Republic	-Determine calcidiol levels in a group of autistic children and compared with healthy children of the same age as controls	45ASD 100% male 40 CG	4–7 years	N/A	-Vitamin D levels	-No significant differences were found between ASD and control groups. ASD: 65.22 ± 25.950; CG: 64.46 ± 20.7
(Guo et al., 2019) China	-Investigate vitamin A (VA) and vitamin D (VD) levels in children with autism spectrum disorders (ASD) -Determine whether VA and VD co-deficiency exacerbates clinical symptoms	332 ASD 48.5% male 197 CG	3–6 years	N/A	-Vitamin D levels	-Vitamin D levels were lower in the ASD group than in CG -Differences in vitamin A and vitamin D accentuated the symptoms of ASD
(Şengenç et al., 2020) Turkey	-Investigate the relationship between autism spectrum disorder (ASD) and vitamin D levels in children and adolescents	100 ASD 100 CG 1529 ASD 80% male	3–18 years	N/A	-Vitamin D levels	-Lower vitamin D levels in ASD group than in CG. ASD100: 42 ± 19.8; ASD1529: 44.60 ± 18.62; CG: 48.57 ± 22.36
(Petruzzelli et al., 2020) Italy	-Evaluate the serum concentration of 25-hydroxyvitamin D (25 (OH) D) in children with ASD (ASD group, n = 54) compared to children affected by other neurological and psychiatric disorders (group without ASD, n = 36)	54 ASD 81.5% male 36 CG	< 18 years	2014–2018	-Vitamin D levels	-The ASD group showed significant levels lower than the CG. ASD: 18.61 ± 8.33; CG: 24.62 ± 13.18
(Alzghoul et al., 2020) China	-Assess the correlation between vitamin D deficiency and autism spectrum disorder in Jordan	83 ASD 100% male 106 CG	< 8 years	N/A	-Vitamin D levels	-A relationship is established between low vitamin D levels and the onset of ASD. The ASD group had more gastro-intestinal complaints. ASD: 23.4; CG: 37.5
(Zhu et al., 2020) China	-Compare the nutritional status and symptoms of preschoolers with autism spectrum disorder from two regions of China -Analyze the association between nutritional status and symptoms of ASD	738 ASD 85.15% male 302 CG	2–6 years	N/A	-Vitamin D levels	-The ASD group presents a higher risk of nutrient deficiencies than the CG group; low levels of vitamin D and folate were associated with worsening ASD symptoms and the development of ASD

*ASD* autism spectrum disorder, *CG*: healthy control group, *ADHD*: Attention-deficit/hyperactivity disorder, *PUFA*: polyunsaturated fatty acids, *DHA*: docosahexaenoic acid, *EPA*: eicosapentaenoic acid, *AA*: arachidonic acid

The studies that provided data on means and standard deviation were included in a meta-analysis. The effects of the studies were compared by estimating the standardized mean differences (SMD) in the score of vitamin D and folate levels among children diagnosed with ASD (ASD group; AG) and children who were not diagnosed (control group; CG). The differences between the SG and the CG for each comparison were grouped to obtain the estimation of the total effect. Statistical models of random effects were applied, considered to be more appropriate for the integration of the results of empirical studies due to the variability they usually present (Sánchez-Meca & Marín-Martínez, 2010), which was subsequently corroborated by the corresponding diagnoses of heterogeneity.

To determine the influence of each of the studies on the overall estimate of the effect, a sensitivity analysis was performed. For each comparison, the heterogeneity of the results was calculated using the chi-square test with a significance level of 0.05, and the I^2^ index was also calculated. For cases where heterogeneity was significant, a subgroup analysis was carried out. Finally, we included in the analysis a study of publication bias to determine whether this could be a threat to the validity of the results of the meta-analysis. RevMan 5.3 was used for the calculations (Cochrane Center).

## Results

**Fig. 1:**
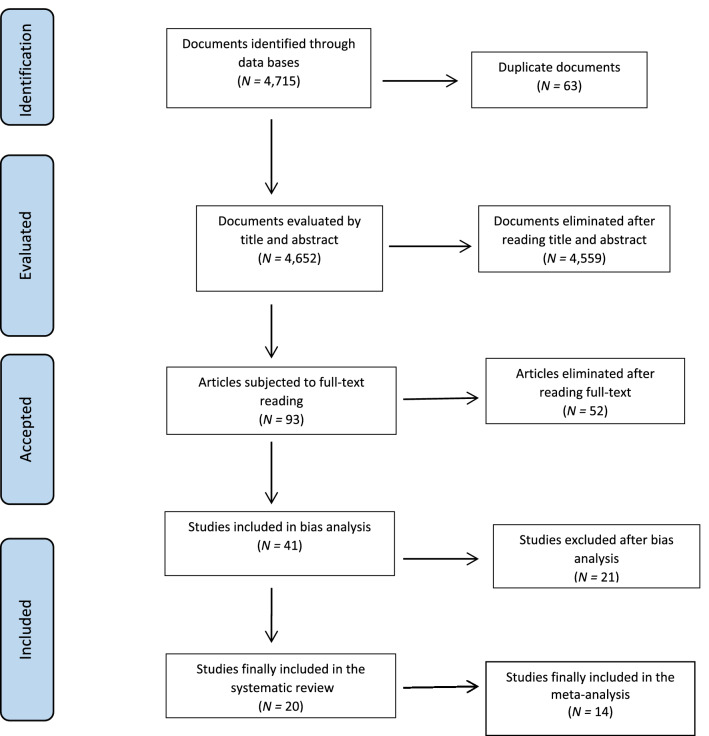
The selection process following PRISMA guidelines (Page et al., 2021)

As shown in Fig. 1, the electronic search initially located 4.715 publications, of which 63 were excluded because they were duplicate documents. After reading the title and abstract, 4559 studies were excluded as they did not respond to the objectives of the meta-analysis or to the research question formulated according to the PRISMA criteria. In the second phase of selection, after full-text reading, 52 articles were excluded for not meeting the inclusion criteria established in the check-lists. Subsequently, after performing the risk-of-bias analysis, 21 studies were excluded for failing to meet the established methodological quality criteria. The scores given by each reviewer to each of the accepted studies, as well as the final score obtained by consensus, are available online, in a document annexed to the protocol.

Finally, 20 articles were included in the narrative review, all of which were quantitative observational studies. Except for one study that analyzed fatty acid (Parletta et al., 2016) levels, in the rest, measurements of vitamin D and folate levels were performed in a sample of children with ASD (*N* = 4171) and compared to a control group of healthy children or children not diagnosed as ASD (*N* = 2982). The intraclass correlation coefficient showed high interjudge reliability (*ICC* = 0.989, *p* < 0.001).

Of the 20 selected studies, five provided incomplete data on or directly did not provide means and standard deviations (Farid et al., 2016; Guo et al., 2019; Liu et al., 2016; Wu et al., 2018; Zhu et al., 2020). The authors of these studies were contacted, but as we received no reply, we decided to exclude them from the meta-analysis. In addition, the study of Parletta et al. (2016) was excluded from the quantitative synthesis because it was the only one offering data on fatty acids. However, the 6 studies mentioned were included in the narrative synthesis due to the interest of their results. Finally, 14 of the reviewed studies that reported vitamin D and folate levels were included in the meta-analysis.

### Studies Included in the Narrative Review

The 20 quantitative studies included were published between 2014 and 2020. The study sample has the following characteristics: the age range is between 0 and 18 years, the minimum and maximum sample sizes in the ASD group were 16 and 1529, respectively, and in the control group, they were 25 and 1240. As for the sex of the sample, it was mostly male (Table 1) and the design of the studies was entirely quantitative and observational.

Three categories were established that correspond to the main objective of our study: vitamin D levels, folate levels, and blood levels of fatty acids.

#### Vitamin D Levels

Of the 20 studies included in the review, 19 papers provide data on vitamin D levels. As can be seen in Table 1, in 16 articles out of the total included in our work, vitamin D levels are shown to be lower in subjects belonging to the ASD group than in subjects in the control group (Altun et al., 2018; Alzghoul et al., 2020; Arastoo et al., 2018; Bala et al., 2016; Bener et al., 2014, 2017; El-Ansary et al., 2018; Farid et al., 2016; Fernell et al., 2015; Garipardic et al., 2017; Gong et al., 2014; Guo et al., 2019; Petruzzelli et al., 2020; Şengenç et al., 2020; Wu et al., 2018; Zhu et al., 2020).

Moreover, three of the studies analyzed found no significant differences between the blood levels in the two groups (Bičíková et al., 2019; Liu et al., 2016; Uğur & Gürkan, 2014).

#### Folate Levels

Blood levels of folate were measured in five of the selected studies (Altun et al., 2018; Bala et al., 2016; Garipardic et al., 2017; Liu et al., 2016; Uğur & Gürkan, 2014). Altun et al. (2018) achieved significant results in terms of the ASD group’s folate levels compared to the CG; lower blood levels were obtained in subjects in the ASD group than those of healthy control: ASD: 121.16 ± 8.04; Control:172.31 ± 17.19 (M ± SD).

The rest of the work about folate in the selected studies did not obtain significant results for this hematological parameter (Table 1).

#### Fatty Acid Levels

Only one study was included in this review that offered data on fatty acid levels and met methodological quality criteria (Parletta et al., 2016). In this study, in a sample of 85 children with ASD and 401 with Attention Deficit Hyperactivity Disorder (ADHD) compared to 79 of the control group, the authors analyzed the blood levels of DHA, AA, EPA + DHA, finding lower values in the ASD and ADHD group compared to the CG of healthy subjects (Table 1).

### Studies Included in the Meta-Analysis

Meta-analysis was performed for vitamin D and folate levels of 14 of the previously selected studies (Altun et al., 2018; Alzghoul et al., 2020; Arastoo et al., 2018; Bala et al., 2016; Bener et al., 2014, 2017; Bičíková et al., 2019; El-Ansary et al., 2018; Fernell et al., 2015; Garipardic et al., 2017; Gong et al., 2014; Petruzzelli et al., 2020; Şengenç et al., 2020; Uğur & Gürkan, 2014) to obtain an estimate of the average effect size for a 95% confidence interval. For heterogeneity analysis, the values of χ^2^ and the *I*^2^ index were estimated.

#### Vitamin D Levels

**Fig. 2:**
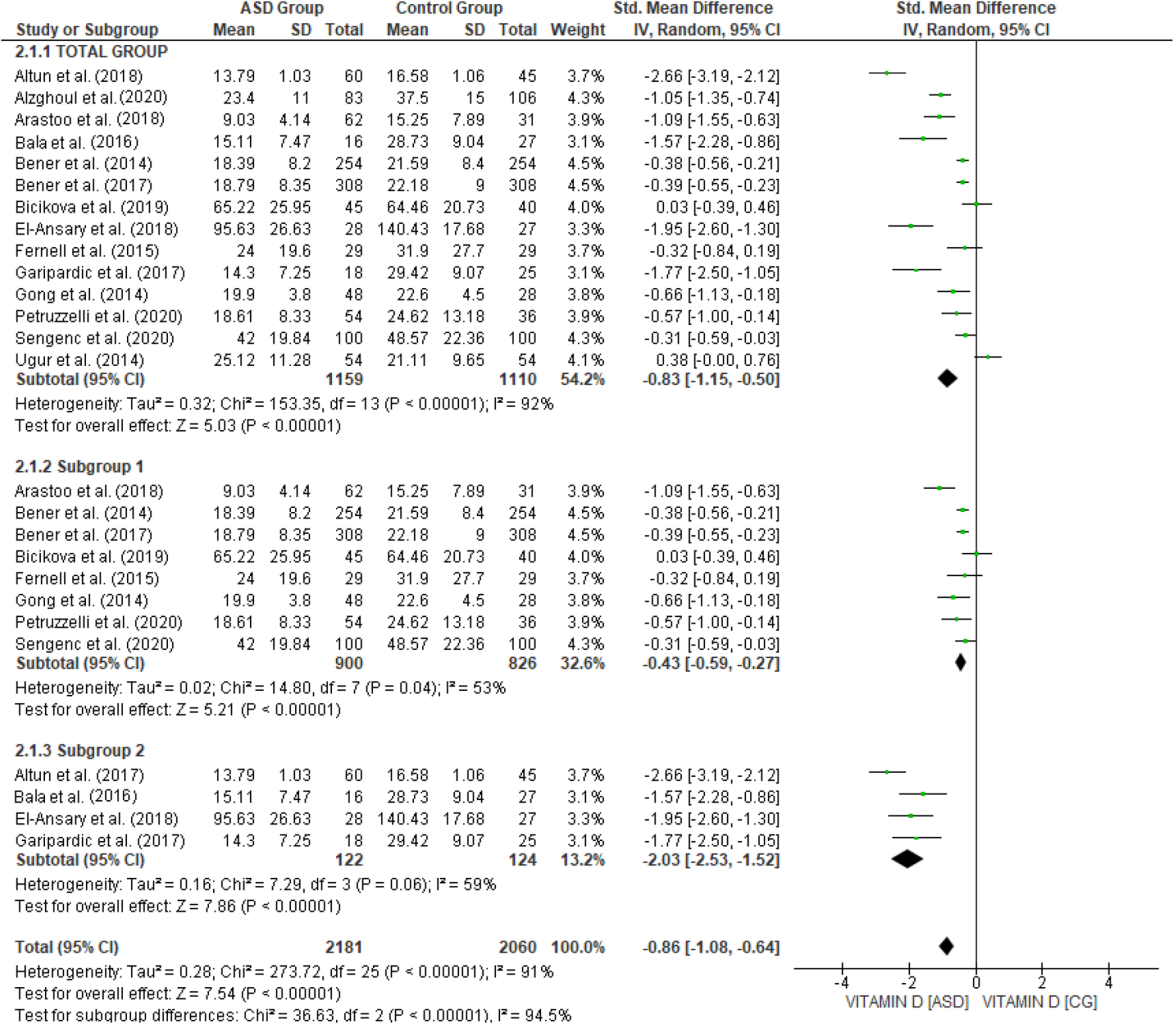
Vitamin D levels ASD Group vs Control Group

The data of 2269 adolescents were available (AG = 1159, CG = 1110), included in the 14 complete trials that measured this variable. Vitamin D levels were lower in the group of children diagnosed with ASD compared to children in the CG: SMD, 95% CI = -0.83 [-1.15, -0.50], with significant heterogeneity (χ^2^ = 153.35, *p* = 0.0001; *I*^2^ = 92%). Given the high heterogeneity observed, a subgroup analysis was performed based on the moderating variables, which did not explain the heterogeneity obtained. A sensitivity analysis was then performed, repeating the calculations by extracting the studies one at a time. This second analysis established two groups, included in Fig. 2.

The subgroup that included the highest number of studies (8 studies, *N* = 1726; SG = 900, CG = 826) continued to show lower levels of vitamin D in children with ASD, although the average effect size remained small, SMD, 95% CI = − 0.43 [− 0.59, − 0.27], observing greater homogeneity between the different studies (χ^2^ = 14.80, *p* = 0.04; *I*^2^ = 53%). The second subgroup (4 studies, *N* = 246; SG = 122; CG = 124) maintained the trend observed in the previous analyses, although it had a larger mean effect size, SMD, 95% CI = − 2.03 [− 2.53, − 1.52], and the heterogeneity analysis was non-significant (χ^2^ = 7.29, *p* = 0.06; *I*^2^ = 53%). Significant differences were observed between the two subgroups (χ^2^ = 36.63, *p* < 0.001; *I*^2^ = 94.5%).

#### Folate Levels

**Fig. 3:**
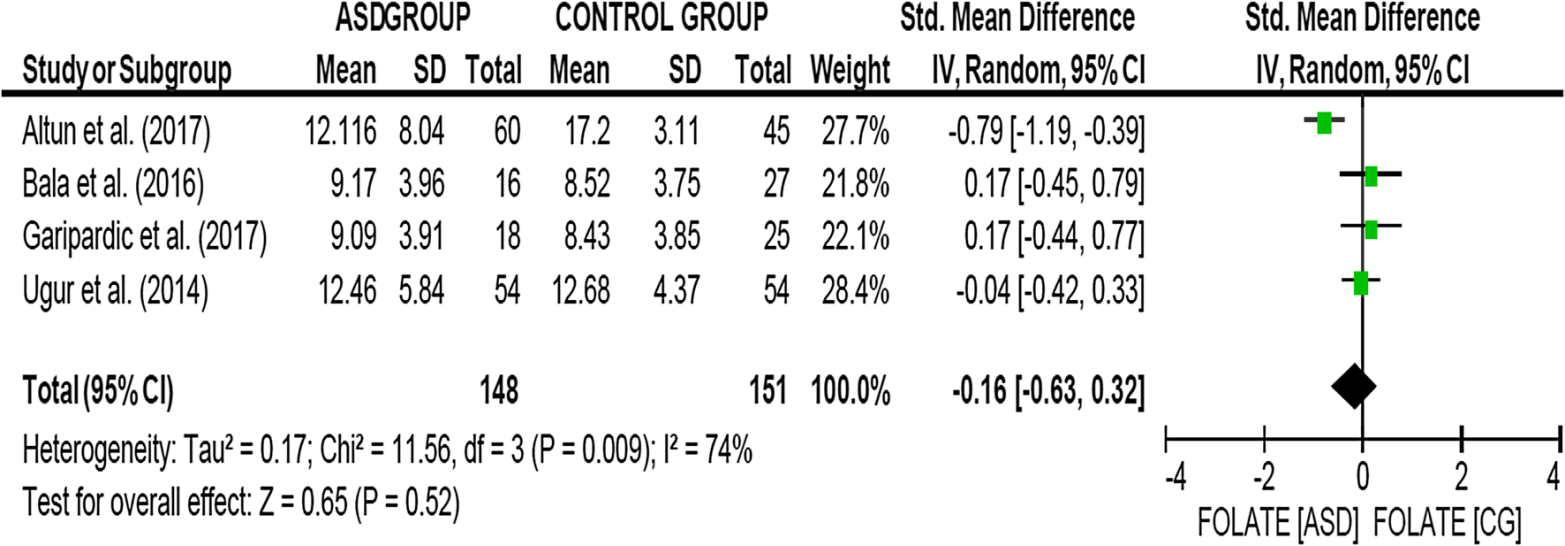
Folate levels ASD Group vs Control Group

As shown in Fig. 3, data on 299 children were available (SG = 148, CG = 151), included in 4 complete observational studies measuring this variable. Folate levels showed no significant differences in the group of children diagnosed with ASD compared to children in the CG: SMD, 95% CI = − 0.16 [− 0.63, 0.32], with significant heterogeneity (*χ*^*2*^ = 11.56, *p* = 0.009; *I*^*2*^ = 76%). Given the scarcity of studies that measured this variable and considering that no significant differences were observed between the two groups, no analyses were carried out to explain this heterogeneity.

### Analysis of Publication Bias

To assess the bias potential for this study, a funnel plot was performed for each of the meta-analyses (Addendum 2). For both measures, the distribution was symmetrical, so there could be no publication bias that would compromise the interpretation of effect sizes found. However, it should be considered that there were few comparisons in the meta-analysis of folate levels, so this type of graph might not be appropriate to detect this possible bias.

## Discussion

The main objective of this work was to study differences in blood levels of vitamins and fatty acids between children diagnosed with ASD compared to children without this diagnosis.

The results obtained in the meta-analysis showed statistically significant differences in the blood levels of vitamin D, which were lower in children diagnosed with ASD than in the CG. The data obtained largely coincide with the meta-analysis of Wang et al. (2016), considered to be the first meta-analysis that investigated a possible relationship between low vitamin D levels and ASD. This work, which included 11 studies published between January 1980 and May 2015, with a total of 870 ASD patients and 782 controls, suggested an increased risk for ASD in children with vitamin D deficiency. Wang et al. (2021), in a recent publication, continued and updated the initial meta-analysis until November 2019; the results obtained are similar to those already published.

Our study extends the time range until December 2020; on another hand, unlike the aforementioned work, this meta-analysis provides a narrative review of all the selected studies (Table 1) and incorporates data on folic acid levels in children. In this sense, to our knowledge, our study is the most up-to-date meta-analysis carried out on blood levels of vitamin D and folate, measured in groups of children diagnosed with ASD and compared to a group of children without this disorder.

The risk of developing an ASD and its relationship to serum vitamin levels is the main goal of many authors in their studies: cause or consequence? remains a key question in the studies analyzed. We find many authors who argue that a vitamin D deficiency can function as a precursor to the development of ASD: for example, Bener et al. (2017) established that anemia and vitamin D deficiency were more present in the ASD group of participants than in the children of the control group, and proposed that this deficiency is a determinant for this disorder. Similarly, Fernell et al. (2015) stated that low vitamin D levels increase the risk of developing ASD. In this line, Ansary et al. (2018) found a significant inverse association between hypovitaminosis D and impaired cognitive development and suggested a possible leading role of vitamin D in the development of ASD.

Following the study of the etiology, other authors have focused on analyzing the nutritional status to obtain data on vitamin levels; for instance, Zhu et al. (2020), who observed a higher risk of nutritional deficiencies in the ASD group than in the control group. Petruzzelli et al. (2020) also argued that vitamin D deficiency may pose a risk factor for developing ASD, whereas Gong et al. (2014) went further, proposing that serum levels of 25 (OH) D could be used as an auxiliary diagnostic tool for early detection of ASD.

On another hand, in terms of the cause, following the initial approach, we highlight authors such as Altun et al. (2018) who, in addition to establishing a relationship with vitamin D deficiency and folate with the development of ASD, argue that children with ASD have lower numbers of vitamin receptors, including those of vitamin D, which favors hypovitaminosis.

As can be seen, the study of the causal relationships of ASD, the etiology, symptoms, and course of the disease are topics about which there is not yet scientific unanimity. As for vitamin supplementation to correct the deficit, many authors have conducted experimental studies, providing supplements to improve the symptoms of the disease (Bener et al., 2014; Bent et al., 2017; Jia et al., 2015; Song et al., 2020; Wu et al., 2018). Based on the results obtained in our study, we consider that it is necessary to take into account the nutritional status of patients diagnosed with ASD and, therefore, vitamin and fatty acid levels, as these elements are involved in the proper functioning of our body and are key pieces of neurodevelopment.

We could not analyze gender differences in our study given the majority proportion of the male sex in all studies included, some of which included only males in the group of participants diagnosed with ASD (Alzghoul et al., 2020; Bičíková et al., 2019; El-Ansary et al., 2018). According to data provided by the CDC, ASD is diagnosed in all racial, ethnic, and socioeconomic groups, but it is 4.5 times more common in boys than in girls. Similarly, DSM-V (APA., 2014) establishes a 4:1 relationship in favor of male onset.

Moreover, we found three studies that do not establish a relationship between vitamin D and/or folate levels with cases of ASD (Bičíková et al., 2019; Liu et al., 2016; Uğur & Gürkan, 2014). Causes that might explain these discrepancies with our results could include the sample size and the season of the year when the blood sample was obtained. Thus, we found that in the study of Ugür and Gürkan (2014), they took into account the months of higher and lower sun exposure, unlike the other two studies.

Meta-analyzed data of folate levels in groups of children with ASD showed no significant differences with the controls. However, we found other meta-analyses focused on the association between folate supplementation during pregnancy and the onset of autism (Iglesias et al., 2019; Wang et al., 2017). In the study, Iglesias et al. (2019) found favorable results for the association between reducing the occurrence of new cases of autism, specifically by 58%, with folate supplementation during the prenatal period. Likewise, Wang et al. (2017) achieved similar results, suggesting a reduction in the risk of developing autism in mothers supplemented with folic acid during pregnancy.

We cannot conclude the relationship of fatty acid levels with ASD, as only one study was selected to analyze these levels (Parletta et al., 2016), although there are some indications in this sense. In fact, some studies have carried out fatty acid supplementation interventions, obtaining favorable results for the clinical symptoms of children with ASD, with improved symptoms of irritability, hyperactivity, and social function (Mazahery et al., 2016; Ooi et al., 2015).

Finally, we believe that it is necessary to continue research in this field with larger samples that examine causal relationships for etiological risk factors, as well as works clarifying the causes of vitamin D deficiency in children diagnosed with autism, and the benefits of supplementation.

## Conclusions

The studies contained in this review and the meta-analyzed data allow us to establish the following conclusions, following the proposed objectives.

First, our study has reported vitamin D deficiency in ASD child samples compared to a child control group without this disorder. Likewise, our meta-analysis for this parameter corroborates this fact (Fig. 2).

Regarding blood values of folate and fatty acids, we could not establish significant relationships between a deficit and ASD. More specific studies are needed for these biochemical parameters to indicate these relationships.

## Limitations and Strengths

As in any study of this nature, there is a potential selection bias, which we attempted to avoid by searching and selecting peers as described in the methodological section. The potential publication bias has been taken into account by interpreting the respective funnel plot. On another hand, we found significant heterogeneity among the studies, so subgroup analyses were carried out, the results of which were offered to provide all the necessary information to the reader. However, given the scarcity of studies included in some cases, the results should be considered with caution.

## Implications for Future Research and Clinical Practice

The results indicate the importance of studying vitamin D and folate supplementation in the treatment of autism. In fact, numerous clinical trials whose main intervention was vitamin D supplementation in children with ASD obtained positive results in the symptomatic improvement of autism. However, there are no direct comparisons for many of these interventions. In this regard, the need for network meta-analyses to assess the overall effect size of these treatments and to establish a hierarchy among them in terms of their effectiveness should be considered. Moreover, some of the studies did not provide data on means and standard deviations or did not use control groups. Similarly, most studies evaluated results regarding vitamin D levels, and to a lesser extent, folate; hardly any studies were found to provide complete data on fatty acids, so they could not be included in the meta-analysis. For subsequent studies, we recommend the use of standard measures and homogeneous instruments, as well as the development of clinical studies that include the complete results of these substances to establish clearer relationships about the roles of vitamin D, folate, and fatty acids with ASD.

Finally, considering that the results presented report lower values of vitamin D in children diagnosed with ASD, it would be pertinent to carry out routine analytical controls of these serum levels in children in whom this disorder is diagnosed or who present compatible symptoms.

## Supplementary Information

Below is the link to the electronic supplementary material.

Supplementary file1 (DOCX 15 KB)

Supplementary file2 (DOCX 42 KB)
