# Supplementary material for: Serum Vitamin D, Folate and Fatty Acid Levels in Children with Autism Spectrum Disorders: A Systematic Review and Meta-Analysis
Source: J Autism Dev Disord. 2021 Nov 3;52(11):4708–21. doi: 10.1007/s10803-021-05335-8 (PMC9556366; doi:10.1007/s10803-021-05335-8)
Supplement: Supplementary file 1 — Supplementary file1 (DOCX 15 KB) [file 10803_2021_5335_MOESM1_ESM.docx]

**ADDENDUM 1**

*Search strategy*

|  | | | **Pubmed** | | **Medline** | | **PsycInfo** | | **WOS** | | **Cochrane** | |
| --- | --- | --- | --- | --- | --- | --- | --- | --- | --- | --- | --- | --- |
| **#1** | 25-OH[Title/Abstract] AND cholecalcifero[Title/Abstract] OR cholecalciferol[Title/Abstract] OR vitamin[Title/Abstract] AND D3[Title/Abstract] OR vitamin D3[Title/Abstract] AND autistic disorder[Title/Abstract] OR autism[Title/Abstract] OR ASD.[Title/Abstract] | | | TI (autism spectrum disorders or autism or asd) AND TI (vitamin d or ergocalciferol or cholecalciferol or 25#hydroxyvitamin d or vitamins) OR TI (omega-3 fatty acids or omega 3) | | TI (autism spectrum disorders or autism or asd) AND TI (vitamin d or ergocalciferol or cholecalciferol or 25#hydroxyvitamin d or vitamins) OR TI (omega-3 fatty acids or omega 3) | | TEMA: (autism spectrum disorder or autism or autistic disorder or asd) AND TEMA: [vitamin d or ergocalciferol or (25-OH and cholecalciferol)] or [vitamins OR (omega-3 fatty acids or omega 3)] | | TODO EL TEXTO: Autism spectrum disorder or autism or autistic disorder or ASD  TODO EL TEXTO: AND. vitamin d or ergocalciferol or vitamin D3 or cholecalciferol or Vitamins  TODO EL TEXTO. AND. omega-3 fatty acids or omega-3 | |  |
| **Total** | | 1640 | | 2236 | | 254 | | 570 | | 15 | |  |
