# Supplementary figures and images for: Serum Vitamin D, Folate and Fatty Acid Levels in Children with Autism Spectrum Disorders: A Systematic Review and Meta-Analysis
Source: J Autism Dev Disord. 2021 Nov 3;52(11):4708–21. doi: 10.1007/s10803-021-05335-8 (PMC9556366; doi:10.1007/s10803-021-05335-8)

**ADDENDUM 2**

**Funnel Plot**

*Folate Levels*


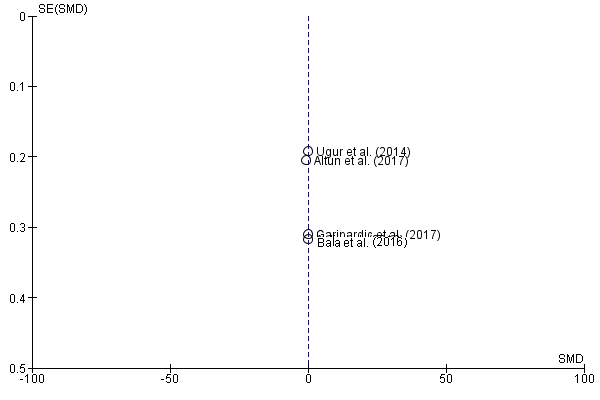


*Vitamin D Levels*

*
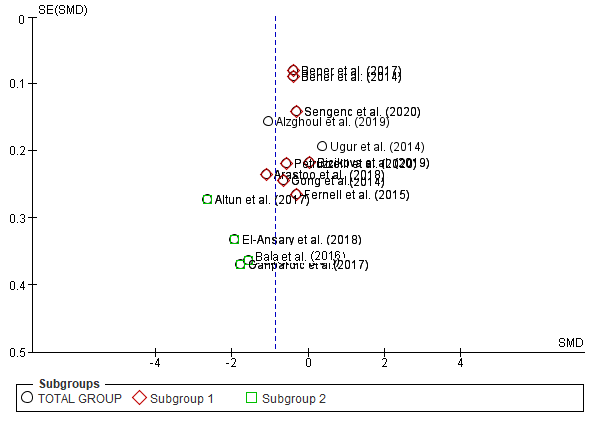
*

Supplement: Supplementary file 2 — Supplementary file2 (DOCX 42 KB) [file 10803_2021_5335_MOESM2_ESM.docx]
